# Sleep quality, sleep quantity, and sleep timing: contrasts in Austrian and U.S. college students

**DOI:** 10.3389/frsle.2024.1487739

**Published:** 2024-11-22

**Authors:** June J. Pilcher, Elizabeth G. Rummel, Claus Lamm

**Affiliations:** ^1^Department of Psychology, Clemson University, Clemson, SC, United States; ^2^Social, Cognitive and Affective Neuroscience Unit, Department of Cognition, Emotion, and Methods in Psychology, Basic Psychological Research and Research Methods, Faculty of Psychology, University of Vienna, Vienna, Austria

**Keywords:** sleep habits, timing of sleep, sleep duration, cross-national, cross-culture, environment

## Abstract

**Objective:**

The current study compared self-reported sleep in undergraduate students in Austria and the United States.

**Methods:**

The Pittsburgh Sleep Quality Index responses from 292 Austrian university students (237 females) and 313 U.S. university students (189 females) were analyzed. In addition to the standard scoring procedure for the scale and the individual components of the scale, the data were also evaluated as separate sleep quality and sleep quantity factors using ANOVAs. Sleep timing (bedtime, wake-time, and midpoint) was also examined using independent *t*-tests.

**Results:**

Austrian students reported better sleep quality and quantity than the U.S. students. In addition, Austrian students had more sleep disturbances and took longer to fall asleep but slept longer and used less sleep medication than U.S. students. Austrian students also went to bed earlier and woke up later than U.S. students.

**Conclusions:**

The current results indicate that sleep in undergraduate students varies across countries. A major difference between the two groups of students was the university setting with the Austrian students living in a large urban area and the U.S. students living in a rural college town, suggesting that the environment could impact student sleep and sleep choices. Finally, the current data indicate that examining sleep quality as a separate factor from sleep quantity provides additional information about sleep in college students. Better documenting sleep and sleep habits in college students across different countries can address important differences that universities and societies can use to help improve sleep and wellbeing in their students.

## 1 Introduction

Maintaining good sleep habits is one of the most essential health-related decisions that humans make each day. College students are particularly susceptible to poor sleep-related choices due to a combination of academic demands, social aspirations, a tendency to delay sleep onset, and easy access to technology. Furthermore, disturbed sleep negatively impacts self-control (Pilcher et al., 2015), perhaps making good sleep choices even more difficult for college students. Research suggests that college students are, indeed, prone to sleep deprivation, poor sleep quality, and poor sleep habits (Hershner and Chervin, 2014; Wang and Bíró, 2021). As such, they provide a population in which variability in sleep and sleep habits can be studied across different settings.

Sleep is often viewed as having two major components: sleep quality and quantity. Although many studies conclude that both sleep quality and quantity are important for many aspects of daily life including health, cognitive functioning, and social interactions (Barnes and Drake, 2015), a recent review study concluded that sleep quality could be more important than sleep quantity (Kohyama, 2021). It is also possible that sleep quality and quantity interact in their effects. For example, in college students sleeping about 7 h a night, sleep quality was better related to subjective health, affect, and mood than sleep quantity (Pilcher et al., 1997). Similarly, medical students reporting adequate sleep quantity still report issues with sleep quality and daytime sleepiness (Ayala et al., 2017; Azad et al., 2015). As such, sleep quality could be the more important factor when experiencing adequate sleep duration, but sleep quantity could be the larger concern when individuals experience partial or total sleep deprivation.

Another feature of good sleep habits is the timing of sleep. Many people experience irregular sleep times (Takasu et al., 2012) which can lead to long-term negative health effects (Chaput et al., 2020) and increasing probability of sleep loss. Research indicates that college students report changes in sleep timing, sleep quality, and sleep quantity during their college years (Galambos et al., 2013). In addition, sleep timing is related to both sleep quality and quantity in that improving sleep hygiene in large part by stabilizing sleep times can improve both quality and quantity of sleep (Knufinke et al., 2018).

The widely used Pittsburgh Sleep Quality Index (PSQI) (Buysse et al., 1989), provides measures of each of these aspects of sleep: sleep quality, sleep quantity, and sleep timing. The original scoring paradigm for the PSQI resulted in one sleep quality score, suggesting that the combination of different aspects of sleep creates a better estimate of sleep. However, studies have used a factor analysis approach to examine PSQI data to determine if the data could be better viewed as more than the single factor that the PSQI originally advanced. For example, one multi-national study concluded that the single-factor interpretation of the PSQI might not best characterize sleep and suggested that the best fit was a two-factor model representing sleep quality and sleep quantity (Gelaye et al., 2014). Although there is some variability in the number of factors found in the PSQI data across different studies using a factor analysis approach (Liu et al., 2021), the larger number of studies identify just two factors, sleep quality and quantity (Manzar et al., 2018). Furthermore, even the studies finding more than two factors in the PSQI, identify sleep quality and sleep quantity as two of those factors (Guo et al., 2016; Liu et al., 2021; Magee et al., 2008). As such, based on the parsimony principle, using a two-factor approach to PSQI data representing sleep quality and quantity provides an additional means of examining PSQI data.

Because sleep habits are shaped by cultural norms (Airhihenbuwa et al., 2016; Williams et al., 2015), it is important to examine sleep across different nationalities. Sleep quantity has received more attention than either sleep quality or sleep timing in multi-national studies. Previous research indicates that sleep duration differs across multiple cultures (Cheung et al., 2021; Olds et al., 2010; Peltzer and Pengpid, 2016; Steptoe et al., 2006; Walch et al., 2016), however, the evidence is mixed with no easily discerned pattern. For example, one study found that Japanese college students slept less than Bulgarian college students (Steptoe et al., 2006). A review study found less sleep duration reported in Asian countries and greater sleep duration reported in European countries with North American countries in the middle (Olds et al., 2010). Another study found less sleep reported by college students from Southeast Asia and sub-Saharan African countries and the most sleep reported by students from south Asian countries and China (Peltzer and Pengpid, 2016). In contrast, few studies have examined sleep quality across countries particularly in adolescence and college-aged adults. One study found better sleep quality in Italian adolescents than U.S. adolescents (LeBourgeois et al., 2005) while another study found better sleep quality in college students in Turkey and the worst sleep quality in college students in Hungary with students in Greece and Poland in the middle (Lesińska-Sawicka and Nagórska, 2022). Finally, although the timing of sleep is particularly affected by different cultures where some cultures have delayed sleep onset times while other cultures do not (Kuula et al., 2019), little research has compared sleep times across different nationalities in adolescents and young adults.

The purpose of the current study is to examine sleep quality, sleep quantity, and sleep timing in convenience samples of Austrian and U.S. college students. It must be noted that there is little research examining sleep habits in college students across different countries, however, we developed and tested the following hypotheses. (1) Austrian undergraduates would report better sleep quality than U.S. undergraduates. (2) Austrian undergraduates would report greater sleep quantity than U.S. undergraduates. Because of the sparse literature, we could not develop a meaningful hypothesis for sleep timing.

## 2 Methods

### 2.1 Study 1: Austria

#### 2.1.1 Participants

A total of 292 Austrian university students participated in the study (237 females; mean age = 21.73 years; SD = 2.36 years). Participants were solicited through their classes and an online platform and received course credit upon completion of the study. The data for this study were made available for the current analyses in a de-identified form and approved for use by the U.S. university internal review board but did not require an official approval form. Informed consent was obtained from all participants prior to the start of the study.

#### 2.1.2 Procedures

Participants completed an online series of self-report surveys and short tasks on sleep, affect, and emotional regulation. All measures that were not available in German were translated from English to German and then back-translated to English to ensure accuracy. The battery took ~50 min to complete and was available on weekdays from 11:00 to 15:00 for 4 weeks during the spring semester.

### 2.2 Study 2: United States

#### 2.2.1 Participants

A total of 313 students at a university in the southeastern United States participated in the study (189 females; mean age = 18.42 years; SD = 1.29 years). Participants were recruited through an undergraduate introductory psychology course and received course credit upon completion of the study. The study was approved by the university institutional review board. Informed consent was obtained from all participants prior to the start of the study.

#### 2.2.2 Procedure

Participants completed an online set of surveys assessing sleep habits, sleepiness, and mood. The surveys took approximately 20 min to complete and were available on weekdays between 11:00 and 15:00 for 2 weeks during the spring semester.

### 2.3 Measures

The Pittsburgh Sleep Quality Index (PSQI) was completed by both the Austrian and U.S. participants. The PSQI (Buysse et al., 1989) is a 19-item survey that evaluates sleep-related variables during the past month. The PSQI provides seven sleep-related components: sleep disturbances, sleep latency, subjective sleep quality, sleep duration, habitual sleep efficiency, use of sleep medication, and daytime dysfunction. Each component is scored from 0 to 3 with 3 indicating more dysfunction. The components can then be summed to produce a global sleep quality score ranging from 0 to 21 with higher numbers indicating poorer sleep quality. The PSQI has been validated in several studies (Carpenter and Andrykowski, 1998; de la Vega et al., 2015) and has been widely used in clinical and non-clinical populations, as well as many research settings (Liu et al., 2021). The German version of the PSQI has also been successfully used in German adults (Hinz et al., 2017). The Cronbach alpha was calculated for each of the samples used in the current study. The Austrian sample had a Cronbach alpha of 0.78. The U.S. sample had a Cronbach alpha of 0.69.

### 2.4 Data analyses

All data analyses were completed using SPSS version 27 (SPSS Inc., Chicago, IL). We followed the standard scoring protocol for the PSQI (Buysse et al., 1989) to calculate scores for the seven components of the PSQI and the PSQI global score. We then completed a 2 × 8 ANOVA to examine the differences between the Austrian and U.S. participants for the PSQI-based scores.

In addition to the standard scoring protocol for the PSQI, we calculated two factors, sleep quality and sleep quantity, based on the factor analysis reported by Gelaye et al. (2014). The sleep quality factor averaged the sleep disturbances, sleep latency, daytime dysfunction, sleep quality, and sleep medication components. The sleep quantity factor averaged the sleep duration and sleep efficiency components. Using a 2 × 2 ANOVA, we examined potential differences between the Austrian and U.S. students for the sleep quality and sleep quantity factors.

Based on the information reported in the PSQI, we also calculated the midpoint of sleep for both groups of undergraduates. We then compared bedtime, wake-time, and midpoint of sleep for Austrian and U.S. students using independent *t*-tests.

## 3 Results

The descriptive data for all PSQI-related variables are shown in Table 1. The ANOVA results indicate that Austrian students had more sleep disturbances, *F*_(1, 603)_ = 17.66, *p* < 0.001, $\eta_{p}^{2}=0.029$, and a longer sleep latency, *F*_(1, 603)_ = 18.54, *p* < 0.001, $\eta_{p}^{2}=0.030$, than U.S. students. However, Austrian students also had a longer sleep duration, *F*_(1, 603)_ = 48.58, *p* < 0.001, $\eta_{p}^{2}=0.075$, and took fewer sleep medications, *F*_(1, 603)_ = 14.42, *p* < 0.001, $\eta_{p}^{2}=0.023$, than U.S. students. There were also significant differences in the derived sleep quality factor, *F*_(1, 603)_ = 10.18, *p* < 0.001, $\eta_{p}^{2}=0.017$, and sleep quantity factor, *F*_(1, 603)_ = 24.67, *p* < 0.001, $\eta_{p}^{2}=0.038$, with Austrian students reporting better sleep quality and greater sleep quantity than U.S. students. There were no significant differences in the PSQI global score as well as the sleep quality, sleep efficiency, and daytime dysfunction PSQI component scores between the two groups (*p* > 0.05).

**Table 1:** Means and standard deviations for all PSQI-related measures for Austrian and U.S. participants.

	**Austria**		**U.S**.	
	**Mean**	**SD**	**Mean**	**SD**
PSQI global score	5.49	2.21	5.57	2.43
**PSQI component scores**				
Sleep disturbances^*^	1.17	0.43	1.02	0.44
Sleep latency^*^	1.35	0.84	1.04	0.95
Sleep quality	1.13	0.57	1.08	0.58
Sleep duration^*^	0.28	0.50	0.62	0.66
Sleep efficiency	0.31	0.41	0.59	0.79
Sleep medication^*^	0.08	0.37	0.25	0.69
Daytime dysfunction	1.17	0.68	1.15	0.70
**PSQI factor scores**				
Sleep quality factor^*1^	0.98	0.35	0.91	0.38
Sleep quantity factor^*2^	0.30	0.46	0.51	0.61

^*^p < 0.001; 1: average of PSQI components sleep disturbances, sleep latency, daytime dysfunction, sleep quality, and sleep medication; 2: average of PSQI components sleep duration and sleep efficiency.

The average bedtimes, wake-times, and midpoint of sleep times are shown in Table 2. Independent *t*-tests indicate that the Austrian students went to bed significantly earlier, *t*_(603)_ = 6.275, *p* < 0.001, and woke significantly later, *t*_(603)_ = –3.439, *p* < 0.001, than U.S. students. There was no significant difference in the midpoint of sleep times between the two groups (*p*> 0.05).

**Table 2:** Average bedtime, wake-time, and sleep midpoint time with standard deviations for Austrian and U.S. participants.

	**Austria**		**U.S**.	
	**Time**	**SD (hr)**	**Time**	**SD (hr)**
Bedtime^*^	23:39	1.26	00:35	1.03
Wake-time^*^	08:41	1.31	08:20	1.23
Midpoint	04:21	1.14	04:27	0.98

^*^p < 0.001.

## 4 Discussion

The current results indicate that Austrian undergraduates reported better overall sleep than U.S. undergraduates. Although there was no difference in the global PSQI score, the PSQI factor scoring showed that Austrian students had better sleep quality and quantity than U.S. students. There were also differences in some of the PSQI component scores with the Austrian undergraduates reporting more sleep disturbances and a longer sleep latency but also a longer sleep duration and fewer sleep medications than U.S. undergraduates. In addition, on average, Austrian students went to bed significantly earlier and woke significantly later than U.S. students, however, the midpoint of the sleep period was not different between the two groups.

The PSQI factor scoring indicated that the Austrian students reported better sleep quality than the U.S. students thus supporting our first hypothesis. This finding contrasts with the global PSQI score, where no difference was found, suggesting that the global PSQI score represents a different metric when defining sleep quality and supports the use of a factor approach with PSQI data to better identify the multidimensionality of sleep. These results add to the limited literature examining sleep quality in adolescents and young adults (Jahrami et al., 2020; LeBourgeois et al., 2005; Lesińska-Sawicka and Nagórska, 2022) across different countries. Given that sleep quality is influenced by many lifestyle, mental health, social, and physical factors (Wang and Bíró, 2021) all of which could vary across different cultures, additional research is needed.

The longer sleep duration component and the PSQI factor scoring indicated that the Austrian undergraduates reported more sleep quantity than U.S. undergraduates thus supporting our second hypothesis. The current results add to the existing literature on sleep quantity across different countries and support an earlier review study indicating that reported sleep duration is longer in European countries than in North American countries (Olds et al., 2010). However, collapsing across numerous European countries could be problematical given that many of the countries could have differing cultural expectations of sleep habits. Although more research addresses sleep quantity across nationalities than sleep quality or sleep timing, there is not yet a clear pattern or explanation for the differences found across countries.

Our study also found differences in bedtime and wake-time as well as several of the PSQI component scores between the Austrian and U.S. students. The Austrian undergraduates went to bed earlier and stayed in bed longer than the U.S. undergraduates with overlapping times in bed such that the midpoint of sleep did not differ between the two groups. The bedtimes and wake-times reported in the current study for both the Austrian and U.S. undergraduates are generally similar to the times reported for students in Norway (Friborg et al., 2012) and Canada (Galambos et al., 2013). However, students in Ghana went to bed and got up earlier (Friborg et al., 2012) while students in Italy went to bed and got up later (Cellini et al., 2020). Together these data suggest that times in bed for undergraduates vary significantly across different nationalities. In addition, it is interesting that the Austrian students reported a longer sleep duration but also more sleep disturbances and a longer sleep latency than the U.S. students. Perhaps the most logical explanation for this difference is the increased use of sleep medications in the U.S. students which could result in fewer sleep disturbances and a shorter sleep latency. Because the PSQI does not delve into the type of medication, we are limited in a more detailed interpretation of these results. Previous research has not reported on the components of the PSQI and only limited studies report the actual time going to bed and getting up for undergraduates when comparing sleep-related data across different countries. Additional research addressing undergraduate student sleep times and the specific components of the PSQI are needed to understand differences across nationalities.

There would have been many similar experiences for the students in Austria and the U.S. For example, both countries have similar economic conditions and exposure to technology. Climatic factors would have been about the same for both groups with similar exposure to sunlight. Perhaps the major potential contributing factor that differs between the two groups in the current study is the environment. The Austrian university is in an urban setting within a large city, whereas the U.S. university is in a rural, small-town setting, with the campus being the focal point of the town. It is feasible that the Austrian students were more often living at home or at some distance from the university and were less immersed in the lives of other students, whereas the U.S. students were more likely living either on or near the campus surrounded by other students. Although not all research agrees, there is evidence suggesting that undergraduates living away from home report more disturbed sleep than undergraduates living at home (Galambos et al., 2013). It is possible that the better and longer sleep reported by the Austrian students could be due to living in an environment that is not constantly filled with distractions from other students. Being exposed to less constant contact with other students could have resulted in a more stable structure to daily life for the Austrian undergraduates than that experienced by U.S. undergraduates who would have continually been around other students.

There are several limitations to the current studies. First, the data were collected from one university in each country. Future studies could include other universities as well as more diverse countries. Second, the current studies did not document potential environmental differences between the two groups such as living situation, proximity to other students, noise levels, or commute distance to campus. Future studies could be designed to assess these types of environmental conditions. Third, the current studies did not assess potential moderator variables such as eating habits and physical activity which could impact sleep. This could be addressed in future studies. Finally, the current studies focused on self-report data from the PSQI. Future studies could implement other subjective measures as well as objective measures such as wearable devices to provide additional information about sleep habits. Despite these limitations, the current study contributes to the existing literature on how sleep and sleep habits vary across different countries and settings, something that is not yet well documented in the literature.

## 5 Conclusion

Because poor sleep habits and sleep loss can often result in decreases in academic performance, impaired mood, decreased health and wellbeing, and drowsy driving (Hershner and Chervin, 2014; Pilcher and Ott, 1998), it is important to better document sleep quality, quantity, and timing in college students. The current study addresses this by implementing a cross-cultural approach and examining sleep in two nationalities of college students. The results indicate that the Austrian students reported better sleep quality and sleep quantity than U.S. students and use less sleep medication than U.S. students. In addition, the Austrian students go to bed earlier and get out of bed later than the U.S. students. These differences could be due at least in part to the environmental conditions of the two groups of students where the Austrian students were in an urban setting and perhaps less immersed with fellow students than the U.S. students who were in a rural college town setting. Finally, the current study suggests that scoring the PSQI as two factors is an effective method of defining quality and quantity of sleep providing better insight into sleep and sleep choices than a single factor approach.

## Data Availability

The raw data supporting the conclusions of this article will be made available by the authors, without undue reservation.
